# Dietary Administration of Banana (*Musa acuminata*) Peel Flour Affects the Growth, Antioxidant Status, Cytokine Responses, and Disease Susceptibility of Rohu,* Labeo rohita*

**DOI:** 10.1155/2016/4086591

**Published:** 2016-05-16

**Authors:** Sib Sankar Giri, Jin Woo Jun, Venkatachalam Sukumaran, Se Chang Park

**Affiliations:** ^1^Laboratory of Aquatic Biomedicine, College of Veterinary Medicine and Research Institute for Veterinary Science, Seoul National University, Seoul 151742, Republic of Korea; ^2^Department of Biotechnology, Periyar Maniammai University, Thanjavur, Tamil Nadu 613403, India

## Abstract

To explore the feasibility of* Musa acuminata* (banana) peels as a feed additive, effects of banana peel flour (BPF) on the growth and immune functions of* Labeo rohita* were evaluated. Diets containing five different concentrations of BPF (0% [basal diet], 1% [B1], 3% [B3], 5% [B5], and 7% [B7]) were fed to the fish (average weight: 15.3 g) for 60 days. The final weight gain and specific growth rate were higher (*P* < 0.05) in the B5 group. The most significant improvements in immune parameters such as lysozyme, alternative complement pathway, leukocyte phagocytic, superoxide dismutase, and catalase activities were observed in the B5 group. However, the B5 group exhibited the lowest malondialdehyde activity. IgM and glutathione peroxidise activities were significantly elevated in the treatment groups, except in B1, after only 30 days of feeding. Of the examined cytokine-related genes, IL-1*β*, TNF-*α*, and HSP70 were upregulated in the head kidney and hepatopancreas, and expressions were generally higher in the B3 and B5 groups. Moreover, B5 group challenged with* Aeromonas hydrophila* 60 days after feeding exhibited the highest survival rate (70%; *P* < 0.05). These results suggest that dietary BPF at 5% could promote growth performance and strengthen immunity in* L. rohita*.

## 1. Introduction

Global aquaculture production is dominated by freshwater fish species (56.4%), especially carp species (71.9%; 24.2 million tons in 2010) [1]. Intensive rearing of aquaculture fish species generates environmental stress to fish, which can increase susceptibility to various pathogens such as viruses, bacteria, fungi, and parasites [2]; this has led to a huge economic loss. The most common and frequently encountered bacterial pathogen in freshwater aquaculture is* Aeromonas hydrophila*, which causes severe damage to carps [3]. Treatment of infections by using antibiotics and chemotherapeutics at the farm level is either prohibited or infeasible because it may result in an increase in drug-resistant pathogens, environmental hazards, and food safety concerns [4]. Therefore, disease management approaches in aquaculture should be based on preventive measures rather than disease control or treatment, as they are likely to be more cost-effective. Currently, use of “immunostimulants” is gaining interest because of their potential in overcoming the limitations of vaccines and probiotics [5].

Fish respond to infectious agents via both specific and nonspecific mechanisms, but they rely primarily on nonspecific immune responses [6]. Therefore, boosting fish innate immunity may be the most promising approach for disease prevention. Plant extracts are known to promote growth; stimulate appetite; enhance tonicity and immunostimulation; facilitate maturation of cultured species; and possess stress reduction, sexual stimulation, and antipathogenic properties in fish [7]. Products of several herbal plants, for example,* Azadirachta indica* [5],* Psidium guajava* [8],* Rheum officinale* [9],* Withania somnifera* [10],* Rehmannia glutinosa* [2],* Ficus carica* polysaccharide [11], emodin [12, 13], and* Achyranthes aspera* [14], have been reported to enhance fish immunity. Moreover, some products of herbal plants have been reported to alter the expression of cytokine- and immune-related genes in fish [2, 8, 11, 14]. They play vital role in host innate immunity and are indispensable for the use and activation of macrophages, neutrophils, and lymphocytes at infection sites for pathogen elimination [15].

Banana (*Musa* spp.) is vital for food security in many tropical and subtropical countries, and they are the most popular fruit in industrialized countries [16]. Banana is the second leading fruit produced after citrus, contributing to approximately 17% of the world's total fruit production; it is cultivated in over 130 countries [17]. Banana peel, which constitutes up to 35% of the ripe fruit, is a household and industrial food waste discarded in large quantities [18]. It is rich in dietary fibre, proteins, essential amino acids, vitamins, polyunsaturated fatty acids, and potassium [19]. Soluble fibres are well known to lower serum cholesterol and help reduce the risk of colon cancer [20]. Bioactive compounds like flavonoids, tannins, phlobatannins, alkaloids, glycosides, anthocyanins, and terpenoids were found in banana peels, and these compounds have been reported to exert various biological and pharmacological effects (antibacterial, antihypertensive, antidiabetic, and anti-inflammatory activities) [21]. Further, antioxidant compounds (e.g., prodelphinidins, polyphenols, catecholamines, and carotenoids) [22] and high amount of micronutrients [23] were found in the peels of genus* Musa*. The presence of various bioactive compounds in banana peels suggests that the peels possess various medicinal properties and may be useful as immunostimulant [24].

The effect of oral immunostimulants on the immune response in aquatic animals is strongly associated with the dose and duration of application. In the present study, we aimed to investigate the effects of dietary supplementation of banana peels on the growth, antioxidant status, immune parameters, and cytokine gene expression of* L. rohita* and its susceptibility to* A. hydrophila* infection. We also explored the potential of banana peel as a feed additive in aquaculture.

## 2. Materials and Methods

### 2.1. Diet Preparation

Bananas (*Musa acuminata*) were purchased from the local market (Thanjavur, Tamil Nadu) with a skin colour index of 6 (all yellow) [27]. The bananas were rinsed thoroughly in tap water, followed by double distilled water and acetone, to remove any contaminants. They were then separated into pulps and peels. The peels were cut into pieces of about 5 cm^2^ by using a kitchen knife and air dried in the oven at 50°C for 24 h. Then, the pieces were ground into a fine powder by using the laboratory grinder. The obtained flour, called BPF, was stored at −20°C until use.

The basal diet (Table 1) was prepared using a previously described method [8]. Proximate analysis of the basal diet [28] revealed a composition of 33.1% proteins, 8.9% lipids, and 12% ash. The basal diet was used as the control diet. The basal diet was supplemented with BPF at five concentrations: 0% (control diet), 1% (B1), 3% (B3), 5% (B5), and 7% (B7). All ingredients were blended thoroughly into a mixture and then pelletized, air-dried, ground, and sieved into an appropriate pellet size. The pellets were then stored at −20°C until further use.

### 2.2. Experimental Design

*Labeo rohita* fingerlings (mean bodyweight: 15.3 g) obtained from local fish farm (Thanjavur, Tamil Nadu, India) were acclimatised to laboratory conditions in 500 L plastic tanks at 26 ± 2°C for 2 weeks and fed the basal diet. Approximately 20% of the water in all tanks was exchanged daily, and 100% of the water was exchanged every week. Basic physiochemical parameters of the water were measured every week [29]. Oxygen and ammonia concentrations were 6.1–7.3 *μ*g mL^−1^ and 0.03–0.06 *μ*g mL^−1^, respectively, and pH ranged from 7 to 8 throughout the study period.

The fish were randomly divided into five experimental groups, with three replicates in each group. Tank capacity was 200 L, and each tank contained 25 fish (i.e., per group: 25 × 3 = 75 fish). The fish were fed one of the five diets for 60 days, and three times (at 08:00, 13:00, and 18:00 hours) at 3–5% of body weight daily. The amount of feed consumed was determined by daily recovery of excess feed, which was then adjusted every 15 days by batch weighing after 24 h of starvation.

### 2.3. Growth Performance

Ten fish were randomly selected from each tank (i.e., 10 × 3 = 30 fish per group) at day 0, day 30, and day 60 after experimental feeding and batch-weighed to estimate growth performance. Growth performance and survival rate of the fish were calculated using the following formulae: weight gain (WG; g/fish) = *W*_*t*_ − *W*_0_; specific growth rate (SGR) = 100 × [(ln⁡*W*_*t*_ − ln⁡*W*_0_)/*t*]; feed conversion ratio (FCR) = FI/(*W*_*t*_ − *W*_0_), where *W*_*t*_ and *W*_0_ were final and initial weights of the fish, respectively; *t* is the duration of feeding (in days); and FI is feed intake.

### 2.4. Sample Collection

At the end of 30 days and 60 days of experimental feeding, five fish from each tank (i.e., 5 × 3 = 15 fish per group) were collected to study immune and antioxidant parameters. Blood samples were collected by caudal venipuncture with a 1 mL syringe after anesthetizing the fish with diluted MS222 (Sigma-Aldrich, USA). The blood samples were transferred into Eppendorf tubes. After collection, blood was centrifuged at 2000 ×g for 10 min at 4°C. The obtained blood leucocytes and plasma were stored at −20°C for further analysis.

#### 2.4.1. Immune Parameters

*(1) Serum Lysozyme Activity.* Lysozyme activity was measured according to the method described by Ellis [30]. Briefly, the lysozyme substrate was 0.2 mg mL^−1^ of freeze-dried* Micrococcus lysodeikticus* (Sigma, USA) suspension in 0.05 M Phosphate Buffer Solution (PBS), pH 6.2. Serum (100 *μ*L) was added to 1.9 mL of the bacterial suspension and the reduction in absorbance at 530 nm was measured after 0.5 min and 4.5 min at room temperature. Results were expressed in units of lysozyme activity mL^−1^ serum. One unit of lysozyme activity was defined as the amount of enzyme that produces a decrease in absorbance of 0.001 min^−1^ mL^−1^ serum.

*(2) Alternative Complement Pathway (ACP) Activity Assay.* ACP activity was determined and calculated using the method of Yano [31] by using rabbit red blood cells (RaRBC). Briefly, the RaRBC were adjusted to 2 × 10^8^ cells mL^−1^ in 0.01 M ethylene glycol tetraacetic acid–magnesium–gelatin veronal buffer (EGTA–Mg–GVB). The 100% lysis value was obtained by lysing of 100 mL of RaRBC with 3.4 mL of distilled water and measuring the optical density of haemolysate at 414 nm against distilled water. The test serum was diluted and different volumes ranging from 0.1 to 0.25 mL were made up to 0.25 mL total volume before being allowed to react with 0.1 mL of RaRBC in test tubes. After incubation at 20°C for 90 min with occasional shaking, 3.15 mL of a saline solution was added to each tube and centrifuged at 1600 ×g for 10 min at 4°C. The optical density of the supernatant was measured at 414 nm using a spectrophotometer (Beckman Instruments Inc., California, USA). The volume of serum that produces 50% haemolysis (ACH_50_) was determined and the number of ACH_50_ U mL^−1^ was calculated for each group. 

*(3) Leucocyte-Phagocytic Assay. Staphylococcus aureus* was cultured in an agar culture medium for 24 h at 37°C. The cell number was adjusted to 5 × 10^6^ CFU mL^−1^. Phagocytic activity (PA) of the blood leukocytes was determined according to the method described by Cai et al. [32]. Briefly, blood sample was collected into heparinized centrifuge tube and shaken well. Same volume of bacterial suspension was added to the tube. The tubes were then kept at 28°C in a water bath for 30 min and shaken every 10 min. Following incubation, solution was centrifuged at 1500 ×g for 5 min and the precipitate was used further. The upper layer of the precipitate was used to make blood slides. Slides were air dried, fixed with methanol for 3 min, and then stained with Swiss staining solution for 5 min. The number of phagocytotic and unphagocytotic leukocytes was counted under the microscope. PA was expressed as phagocytic rate (PR):

(1) PR%=100×phagocytic leucocytestotal leucocytes−1.

*(4) Immunoglobulin M (IgM) Assay.* Plasma total IgM levels were measured according to the method described by Sharma et al. [10]. Briefly, blood sample was centrifuged for 5 min at 1000 ×g to get clear cells. 0.1 mL of plasma was placed into a serum vial and added 0.1 mL of 12% polyethylene glycol (PEG) that had been suspended in deionised water and incubated at room temperature for 2 h under constant mixing. Following centrifugation at 5000 ×g for 10 min, supernatant was collected and protein concentration was determined. Protein reading from supernatant was the amount of protein taken out by absorption to PEG. Total immunoglobulin was expressed as unit mg mL^−1^. Total immunoglobulin = total protein in individual sample plasma − total protein taken out by absorption to polyethylene glycol.

#### 2.4.2. Antioxidant Parameters

*(1) Superoxide Dismutase (SOD) and Malondialdehyde (MDA) Assay.* SOD activity was determined using an enzymatic assay method with a reagent kit (Randox, Crumlin, UK), as described by Zhang et al. [33]. MDA content was measured using barbituric acid reaction chronometry [34].

*(2) Measurement of Catalase (CAT) Activity and Glutathione Peroxidise (GPx) Activity.* CAT activity was measured by the rate of decrease in H_2_O_2_ absorbance at 240 nm by using a commercially available kit (Sigma-Aldrich, USA). GPx activity was measured using a commercial kit (Ransel RS-504, Randox), according to the manufacturer's instructions. Specific activity was expressed as GPx unit mg^−1^ of total protein.

### 2.5. Expressions of Immune-Related Genes in the Head Kidney and Hepatopancreas

The head kidney and hepatopancreases were dissected from nine fish per group at the end of the feeding trial. Total RNA was extracted from the tissues (kidney and hepatopancreas) by using TRIZOL reagent (Invitrogen, USA), according to the manufacturer's instructions. RNA concentration and purity were analysed using a spectrophotometer (Thermo Scientific, USA), and quality was checked using 1% agarose gel containing 0.5 *μ*g mL^−1^ ethidium bromide. RNA was reverse-transcribed to cDNA by using the SuperScript® cDNA synthesis kit (Life Technologies), according to the manufacturer's instructions. Real-time PCR analysis of* IL-1β*,* IL-10*,* iNOS*,* TNF-α*,* TGF-β*,* HSP70*,* NF-κB*, and a housekeeping gene (*β*-actin) was performed using CFX96*™* Real-Time PCR (Bio-Rad, Laboratories, Inc.), according to standard protocols with primer sequences and thermocycling conditions as indicated in Table 2. To verify the accuracy of each amplicon, melt curve analysis was performed after amplification. All samples were run in parallel with the housekeeping gene to normalize cDNA loading. Gene expression results were analysed using the 2^−ΔΔC_T_^ method after verifying that the primers were amplified with an efficiency of approximately 100% [26], where ΔΔC_T_ = [(C_T_ gene of interest − C_T_ *β*-actin) treatment group − (C_T_ gene of interest − C_T_ *β*-actin) control group]. The C_T_ is defined as the PCR cycle at which the fluorescent signal of the reporter dye crosses an arbitrarily placed threshold.

### 2.6. Challenge Test

Seven-day lethal dose 50 (LD_50_) for* A. hydrophila* MTCC-1739 was 10^7^ CFU/mL, as determined earlier in our laboratory [8], and this strain was reisolated from experimentally infected fish in our laboratory. At the termination of the feeding trial, 10 fish from each tank (i.e., 3 × 10 = 30 fish per group) were selected for the challenge test. The fish (*n* = 30) were injected intraperitoneally with 100 *μ*L of phosphate-buffered saline (PBS) containing 1 × 10^7^ live* A. hydrophila*. For negative control, 10 fish were injected with PBS only. The challenged fish were kept under observation for 14 days and fed the basal diet. Mortality of the fish in each tank was observed over 14 days.

### 2.7. Statistical Analysis

One-way analysis of variance (ANOVA) was used to analyse the data. Multiple comparisons were performed using Tukey's test to analyse the differences between treatments. All statistical analyses were performed using the OriginPro software (version 8; OriginLab Corporation, Northampton, USA). The level of significance was set at *P* < 0.05, and the results were expressed as mean ± SEM.

## 3. Results

### 3.1. Growth Performance

Effects of BPF on the growth performance of* L. rohita* are shown in Table 3. After 30 days of feeding, significantly higher WG and SGR were observed in the B5 group than in the control. However, 30 days of BPF administration had no significant effects on FCR. At the end of the trial, WG and SGR were higher in the treatment groups, and the highest (*P* < 0.05) WG (83.61 ± 1.52 g) and SGR (2.86 ± 0.023 g) values were observed in the B5 group (compared to the control). FCR was significantly lower in the B5 group (4.39 ± 0.02) than in the control.

### 3.2. Immune Parameters

Results of LA and ACP activities are shown in Table 4. Serum lysozyme activity was significantly higher in the B5 group during the whole period of the trial than in the control. ACP activity was significantly higher in the treatment groups, except B1, at any point of time, and it was the highest in the B5 group (Table 4). A slight decline in LA and ACP activities was observed at a higher BPF concentration (i.e., B7) during the trial period.

Effects of dietary administration of BPF on PA and IgM of* L. rohita* are shown in Table 5. After 30 days of feeding, significantly higher PA was observed in the treatment groups, except B1, than in the control. However, PA was significantly higher in the treatment groups at the end of the feeding trial, and the highest activity was recorded in the B5 group (48.70 ± 1.68%). IgM level was significantly higher in the treatment groups, except B1, during the 30 days of feeding. However, 60 days of feeding with BPF did not have a significant effect on IgM levels, although a slight increase in IgM level (compared to the control) was recorded in the B1 group. Surprisingly, IgM levels were lower in the other treatment groups than in the control, and the lowest level (*P* < 0.05) was observed in the B7 group.

### 3.3. Antioxidant Parameters

SOD activity was significantly higher in the B5 and B7 groups than in the control at any point of time (Table 6). The highest SOD activity was observed in the B5 group at any point of time.

After 30 days of feeding, significantly lower MDA activities were observed in the B3, B5, and B7 groups than in the control (Table 6). However, at end of the trial, significantly lower MDA activities were recorded in the B5 and B7 groups. The lowest MDA activity was recorded in the B5 group after 60 days of feeding.

CAT activity was significantly higher in the B5 and B7 groups than in the control (Table 7), and the highest CAT activity was recorded in the B5 group.

GPx activities were significantly higher in the B5 and B7 groups than in the control after 30 days of feeding, and the highest GPx activity was recorded in the B7 group (Table 7).

### 3.4. Gene Expression Analysis

**Figure 1:**
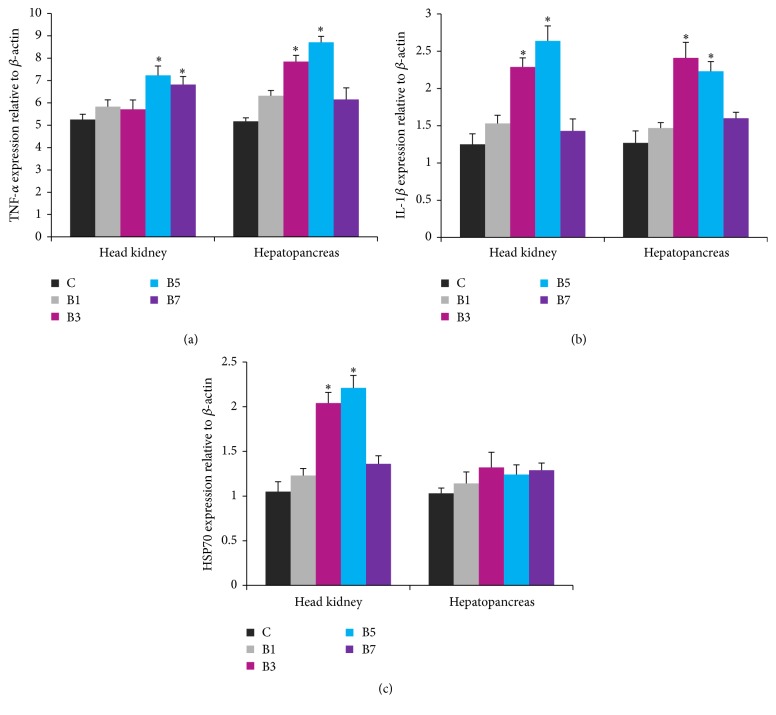
Relative mRNA expressions of upregulated genes (*TNF-α*,* IL-1β*, and* HSP70*) in the head kidney and hepatopancreas of* Labeo rohita* fed banana peel flour (BPF) supplemented diets. A significant difference is denoted by an asterisk (*P* < 0.05). Each bar represents mean ± SEM (*n* = 9).

**Figure 2:**
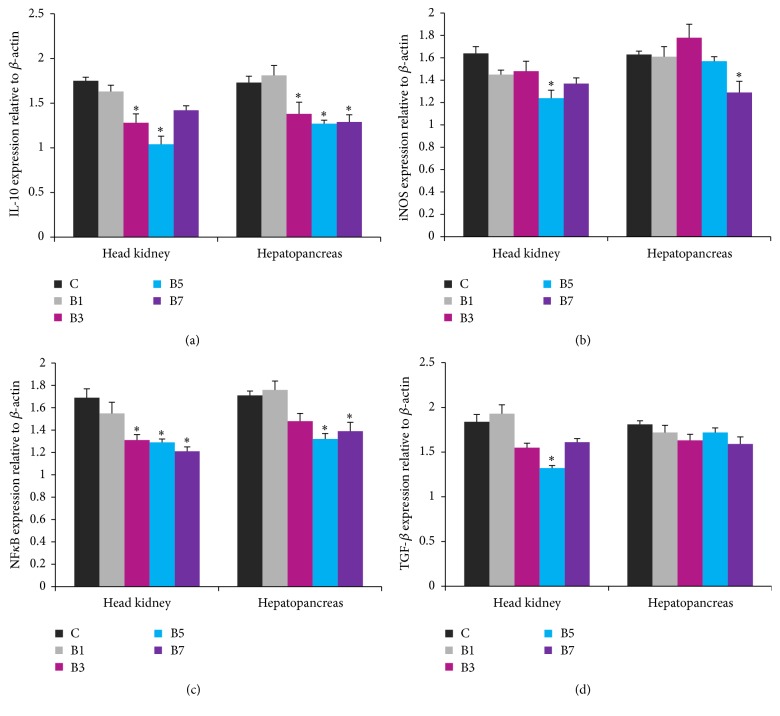
Relative mRNA expressions of* IL-10*,* iNOS*,* NF-κB*, and* TGF-β* in the head kidney and hepatopancreas of* Labeo rohita* fed banana peel flour (BPF) supplemented diets. A significant difference is denoted by an asterisk (*P* < 0.05). Each bar represents mean ± SEM (*n* = 9).

Expression profiles of immune-related genes were examined in the head kidney and hepatopancreas of the fish (*n* = 9) at the end of the feeding trial. Transcription levels of* TNF-α*,* IL-1β*, and* HSP70* were upregulated in the organs of fish fed the experimental diets (Figure 1), whereas expressions of* IL-10*,* iNOS*,* NF-κB*, and* TGF-β* were downregulated (Figure 2).

The B5 group exhibited the highest (*P* < 0.05)* TNF-α* mRNA expression in the head kidney and hepatopancreas tissues (Figure 1(a)).* IL-1β* mRNA expression was the highest (*P* < 0.05) in the head kidneys of the B5 group, followed by B3, whereas* IL-1β* expression was the lowest in the hepatopancreas (Figure 1(b)).* HSP70* expression was significantly higher in the head kidneys of the B3 and B7 groups, and the highest* HSP70* expression was observed in the B5 group (Figure 1(c)). However, dietary treatments had no significant effect on* HSP70* expression in the hepatopancreas.

Effects of BPF on* IL-10*,* iNOS*,* NF-κB*, and* TGFβ* expressions are shown in Figure 2.* IL-10* expression was downregulated in the B3 and B5 groups, and the lowest level was observed in the B5 group (Figure 2(a)). Expression of* iNOS* was downregulated (*P* < 0.05) in the B5 and B7 groups (Figure 2(b)). In addition, a slight increase in* iNOS* expression was observed in the hepatopancreas of the B3 group (Figure 2(b)).* NF-κB* expression was downregulated in both the B5 and B7 groups (Figure 2(c)).* TGF-β* expression was significantly lower in only the head kidney of the B5 group (Figure 2(d)).

### 3.5. Challenge Test

Dietary supplementation of BPF for a longer period enhanced the resistance of* L. rohita* to* A. hydrophila* infection. The highest postchallenge survival (70%) was recorded in the B5 group, whereas the lowest postchallenge survival rate (20%) was observed in the fish fed the basal diet. The B3 group exhibited a survival rate of 56.6%, followed by the B7 (40%) and B1 (26.66%) groups. No mortality was observed in the group injected with PBS alone. Typical symptoms of haemorrhagic septicaemia were observed in moribund or dead fish. Colonies of* A. hydrophila* were isolated from dead fish.

## 4. Discussion

In the present study, dietary supplementation with 5% BPF (i.e., B5) for 60 days significantly increased WG and SGR in* L. rohita*. Recently, we demonstrated that dietary administration of emodin (at 30 mg kg^−1^ of diet) [13] or guava leaves (at 0.5%) [8] for 60 days significantly increased the growth performance of* L. rohita*. Nonprotein energy sources such as carbohydrates and lipids in the diets have the ability to spare dietary proteins by reducing the catabolism of proteins for energy, which can improve retention and ultimately growth in animals [35]. Further, significant reduction in FCR in the B5 group suggested that the fish utilised dietary nutrients more efficiently when feed was supplemented with BPF. However, owing to the complexity of the components used in herbal preparations, the direct cause of growth stimulation is unclear.

A complex system of numerous types of antioxidants (e.g., catalase, glutathione, SOD, and various peroxidases) is present in aquatic animals [9]. SOD, GPx, and CAT are important biochemical parameters and the first line of antioxidant enzymatic defence. Therefore, measurement of these antioxidant parameters may provide a hint of the antioxidant status in fish, and these parameters can serve as biomarkers for oxidative stress [33]. In the present study, supplementation of 5% BPF (i.e., B5) for 60 days resulted in the highest SOD and CAT activities, whereas MDA activity was lowered considerably (*P* < 0.05). In addition, supplementation of BPF significantly enhanced GPx for up to 30 days, and, thereafter, increment was not significant. Similar results were observed in* L. rohita* [13],* Megalobrama terminalis* [33],* Ctenopharyngodon idella* [36], and* Megalobrama amblycephala* [12] after dietary administration with herbal immunostimulants. Our results revealed that BPF at an appropriate concentration could stimulate the secretion of antioxidant enzymes as well as antioxidants, which can efficiently eliminate excess free radicals and regulate the balance of free radical in the body, resulting in improved antioxidant ability [33]. Further, lower MDA content observed in the present study also strengthened the higher antioxidant potential of BPF because MDA has strong biotoxicity and can damage cell structure and function [37]. Bioactive compounds such as phenolic compounds in banana peel may be responsible for the antioxidant activity [22]. Antioxidant potential of banana peels has also been reported in previous studies [22, 24].

As the first line of defence, various peptides such as lysozyme, antibodies, and complement factors inhibit the adhesion and colonization of microorganisms, leading to the prevention of infection and disease [35]. Phagocytosis has also been recognised as an important cellular process in the nonspecific immune system of fish [38]. In the present study, dietary supplementation with 3% (B3) or 5% BPF (B5) for 60 days augmented (*P* < 0.05) serum LA, ACP, and PA in fish, with the highest degree of activity exhibited by the B5 group. Several recent studies have reported that dietary supplementation of plant products enhanced these immune parameters in carp [2, 5, 8, 10, 13], which is consistent with our results. However, higher levels of BPF administration for a longer period had no significant effect on these immune parameters. This is most likely because higher doses of immunostimulants administered over longer periods often lead to immunosuppression, but the exact underlying reason is still unclear.

The predominant antibody type in fish, IgM, is used to identify and neutralize foreign objects such as bacteria and viruses [39]. In the present study, supplementation of 3% to 7% BPF improved (*P* < 0.05) IgM levels in fish up to 30 days, and thereafter the levels gradually decreased. Similar trend of IgM levels has also been reported in previous studies [8, 10, 13]. In line with earlier reports, it can be suggested that stimulation of IgM level is a temporary phenomenon attributable to immunostimulants.

Our study also provides evidence that dietary supplementation with BPF could efficiently regulate the expressions of certain cytokine-related genes in* L. rohita*.* IL-β* and* TNF-α* expression levels are generally considered to be indicators of inflammatory response [40], and they can regulate the production of other cytokines [2]. Coexpression of IL-1*β* and TNF-*α* is not unusual because they share similar roles in the initiation of immune response [41]. We found that dietary administration of 5% BPF (B5) significantly upregulated the expressions of* IL-1β* and* TNF-α* in both examined tissues. Recently, we demonstrated that administration of 0.3% or 0.5% guava leaves for 60 days significantly augmented the expressions of* IL-1β* and* TNF-α* in the head kidney, intestine, and hepatopancreas of* L. rohita* [8]. Dietary administration of* R. glutinosa* augmented the expressions of* IL-1β* and* TNF-α* genes in the common carp,* Cyprinus carpio* [2]. However, abundance of the two genes differed in the same organ of fish that were fed various levels of BPF.

iNOS and nitric oxide (NO) are recognised immunoregulatory factors in the defence against various pathogens in fish [8]. Recently, we observed that dietary administration of guava leaves significantly downregulated the expressions of* iNOS* and* NF-κB* in* L. rohita* [8]. In the present study, we observed that dietary administration of BPF downregulated the expressions of* NF-κB* in the examined tissues, whereas* iNOS* expression was significantly downregulated in only the head kidney in the B5 group. In contrast,* R. glutinosa* root powder enhanced* iNOS* gene expression in the head kidney of* C. carpio* [2]. The mechanism by which BPF downregulated* iNOS* or* NF-κB* expression is not yet known. However, inhibition of NO activity by water extracts of banana peels has been reported in the literature [42].

HSPs are conserved proteins induced by heat and several noxious stimuli, including thermal shock, heavy metals, viruses, oxygen free radicals, and pathological stress [43]. HSP70 has a number of functions, including maintenance of cellular homeostasis, and general protective effects on all living organisms after stress [44]. Liu et al. [9] reported an increase in* HSP70* mRNA expression in* Macrobrachium rosenbergii* after dietary administration of anthraquinone extract for 6 or 8 weeks. However,* HSP70* expression in the blood of grass carp was remarkably downregulated after administration of* Ficus carica* polysaccharide [11]. In this study, expression of* HSP70* was significantly elevated in the head kidney of the B3 and B5 groups, but BPF had no significant effect on* HSP70* expression in the hepatopancreas. Dietary emodin and high doses of vitamin C increased* HSP70* mRNA expression in Wuchang bream under high temperature stress, which is consistent with the results of the present study [12]. However, further studies are necessary to elucidate the mechanism by which BPF affects* HSP70* expression in* L. rohita*.

IL-10 and TGF-*β* are regulatory cytokines with multifunctional roles in the immune system. IL-10 limits the magnitude of immune responses to foreign pathogens [45]. In this study,* IL-10* expression was downregulated in the examined tissues of* L. rohita* fed a B3 or B5 diet. We observed a counter relationship between the expressions of* TNF-α* and* IL-10*. These results correspond with those of previous studies [2, 8, 14]. Swain et al. [46] examined the mechanism of IL-10 induction by blocking NF-*κ*B signalling with BAY11-7082 in kidney cell culture of* Catla catla*. Blocking of NF-*κ*B suppressed IL-10 induction by lipopolysaccharides, suggesting that IL-10 was induced through the NF-*κ*B signalling pathway in* Catla catla*. Normally, TGF-*β* inhibits B-and T-cell propagation and differentiation, antagonises proinflammatory cytokines (*IL-1β*,* TNF-α*, and* IFN-γ*), and blocks expressions of* IL-1β* and* IL-12* receptors [36]. In the present study,* TGF-β* expression was significantly downregulated in the head kidney of the B5 groups alone, which is consistent with the results of previous studies [2, 8]. Active constituents of banana peels may be responsible for observed anti-inflammatory properties. For example, vitamin E, which is present in banana peel, is known to have potent anti-inflammatory activities [47]. Pectin is an eco-friendly and biodegradable polysaccharide present in banana peel and it has been used as an antimicrobial and anti-inflammatory agent [48]. Anti-inflammatory activities of banana peel extracts have also been reported in previous studies [42, 49].

After challenging with* A. hydrophila*, all BPF-fed groups exhibited higher survival than the control, and the highest survival (70%) was observed in the B5 group. Our results indicated that BPF assisted in the control of microbial pathogens as well as infections. Higher survival in B5 fed group may be due to the augmentation of immune parameters (LA, PA, ACP, SOD, and CAT), decreased level of MDA, and stimulation of immune-related genes. Recently, several studies have shown that fish fed with herbal diets exhibited higher resistance to pathogen infections [2, 8, 10, 13, 14, 33]. Active components present in banana peel have higher antibacterial activities against gram-positive and gram-negative pathogens [48, 49]. Moreover, hydroxyapatite nanoparticles derived from banana peel pectin had strong antibacterial activity against* S. aureus* and* Escherichia coli* [48].

## 5. Conclusions

Present study reveals that dietary administration of 5% BPF (B5) for 60 days has the potential to modulate growth parameters, antioxidant status, immune responses, and expression of cytokine genes in* L. rohita*. In addition, 5% BPF upregulated* HSP70* expression and enhanced the postchallenge survival rate of* L. rohita*. Therefore, banana peel can be used as a feed additive in aquaculture to improve fish growth and disease resistance. Therefore, this inexpensive and easily available natural immunostimulant may represent a viable alternative to prophylactic use of chemicals in freshwater aquaculture. However, further studies should be conducted to better understand the mechanisms underlying dietary BPF in fish.

## Figures and Tables

**Table 1:** Formulation and chemical composition of basal diet (g kg^−1^ dry matter).

Ingredients	Concentrations (g kg^−1^)
Ground nut oil cake	390
Rice bran	340
Soybean meal	170
Fish meal	78
Vegitable oil	20
Mineral and vitamin mixture^a^	2

*Proximate analysis *(g kg^−1^)	
Crude protein	331
Crude lipid	89
Ash	120

^a^Every 250 g of mineral-vitamin mixture (Supplevite-M, Sarabhai Zydus Animal Health Ltd., India) provides vitamin A, 500000 IU; vitamin D3, 100000 IU; vitamin B2, 0.2 g; vitamin E, 75 units; vitamin K, 0.1 g; calcium pantothenate, 0.25 g; nicotinamide, 0.1 g; vitamin B12, 0.6 mg; choline chloride, 15 g; calcium, 75 g; manganese, 2.75 g; iodine, 0.1 g; iron, 0.75 g; zinc, 1.5 g; copper, 0.2 g; and cobalt, 0.045 g.

**Table 2:** Real-time primer sequences and thermocycling conditions.

Target gene	Primer sequence (5′ to 3′)	Thermocycling conditions	Reference/accession number
IL-1*β*	ACCCCACAAAACATCGGCCAACC	95°C 30 s, 40 cycles of 95°C 5 s, 61.5°C 30 s, and 72°C 30 s	[25]
	TCTTCTCCATTTCCACCCTCTC		

IL-10	CGCAGTGCAGAAGAGTCGAC	95°C 30 s, 40 cycles of 95°C 5 s, 61.5°C 30 s, and 72°C 30 s	GU256643
	CCCGCTTGAGATCCTGAAATAT		

TNF-*α*	CTCAACAAGTCTCAGAACAATCAGG	95°C 30 s, 40 cycles of 95°C 5 s, 61.5°C 30 s, and 72°C 30 s	[25]
	TCCTGGTTCCTTCTCCAATCTAGCT		

iNOS	GGAGGTACGTCTGCGAGGAGGCT	95°C 30 s, 40 cycles of 95°C 5 s, 61.1°C 30 s, and 72°C 30 s	[8]
	CCAGCGCTGCAAACCTATCATCCA		

TGF-*β*	ACGCTTTATTCCCAACCAAA	95°C 30 s, 40 cycles of 95°C 5 s, 61.5°C 30 s, and 72°C 30 s	AF13694
	GAAATCCTTGCTCTGCCTCA		

NF-*κ*B	TATTCAGTGCGTGAAGAAG	95°C 30 s, 40 cycles of 95°C 5 s, 61.5°C 30 s, and 72°C 30 s	LN590704
	TATTAAAGGGGTTGTTCTGT		

HSP70	GGCAGAAAGTTTGATGACCCA	95°C 30 s, 40 cycles of 95°C 5 s, 61.5°C 30 s, and 72°C 30 s	[26]
	GCAATCTCCTTCATATTCACC		

*β*-actin	AGACCACCTTCAACTCCATCATG	95°C 30 s, 40 cycles of 95°C 5 s, 61.5°C 30 s, and 72°C 30 s	[25]
	TCCGATCCAGACAGAGTATTTACGC		

**Table 3:** Effects of banana peel flour (BPF) on the growth performance of *Labeo rohita*.

Parameters	Control	B1	B3	B5	B7
Initial weight (g)	15.37 ± 0.41	15.62 ± 0.24	15.18 ± 0.68	15.21 ± 0.27	15.6 ± 0.46

0–30 days of feeding					
WG (g)	38.06 ± 0.44^a^	38.65 ± 0.28^ab^	39.24 ± 0.57^ab^	40.97 ± 0.39^b^	39.63 ± 0.48^ab^
SGR	2.94 ± 0.017^a^	2.95 ± 0.041^a^	3.08 ± 0.032^b^	3.14 ± 0.024^cb^	3.03 ± 0.015^ab^
FCR	5.84 ± 0.21	5.83 ± 0.18	5.81 ± 0.29	5.78 ± 0.36	5.81 ± 0.24
Survival (%)	100	100	100	100	100

0–60 days of feeding					
Final weight gain (g)	74.19 ± 1.66^a^	77.06 ± 1.09^ab^	81.42 ± 1.22^b^	83.61 ± 1.52^b^	80.07 ± 1.18^b^
SGR	2.63 ± 0.047^a^	2.66 ± 0.053^ab^	2.78 ± 0.031^b^	2.86 ± 0.023^b^	2.72 ± 0.029^ba^
FCR	4.61 ± 0.03^a^	4.54 ± 0.01^a^	4.48 ± 0.05^ba^	4.36 ± 0.02^b^	4.52 ± 0.03^ba^
Survival (%)	100	98.66	100	100	97.33

Note: WG = weight gain; SGR = specific growth rate; FCR = feed conversion ratio.

Values in the same column with different superscript letters are significantly different (*P* < 0.05). Values are presented as mean ± SEM (*n* = 30 fish in each group).

**Table 4:** Lysozyme (LA) and alternative complement pathway (ACP) activities observed on different sampling days after feeding *Labeo rohita* with banana peel flour (BPF) supplemented diets.

Diet	Immune response			
	LA (unit mL^−1^)		ACP (ACH_50_^b^ unit mL^−1^)	
	30 days	60 days	30 days	60 days
Control	69.3 ± 1.73^a^	71.2 ± 1.64^a^	103.1 ± 2.18^a^	111.4 ± 1.78^a^
B1	71.5 ± 2.3^ab^	74.6 ± 1.91^a^	107.9 ± 2.73^a^	119.1 ± 2.81^a^
B3	76.1 ± 2.64^ab^	82.1 ± 1.1^b^	118.4 ± 3.11^b^	131.7 ± 3.06^c^
B5	81.2 ± 2.51^b^	86.9 ± 1.96^b^	136.7 ± 1.53^c^	143.1 ± 2.21^c^
B7	79.3 ± 1.52^ba^	80.6 ± 0.87^ba^	127.6 ± 2.01^cb^	129.4 ± 1.82^cb^

Values are expressed as mean ± SEM (*n* = 15). Mean values in the same column with different superscript letters vary significantly (*P* < 0.05).

**Table 5:** Phagocytic activity (PA) and immunoglobulin (IgM) activities observed on different sampling days after feeding *Labeo rohita* with banana peel flour (BPF) supplemented diets.

Diet	Immune response			
	PA (%)		IgM (unit mg mL^−1^)	
	30 days	60 days	30 days	60 days
Control	34.37 ± 1.73^a^	36.10 ± 0.64^a^	5.13 ± 0.18^a^	5.42 ± 0.24^a^
B1	36.20 ± 2.1^ab^	39.76 ± 1.27^b^	5.40 ± 0.16^a^	5.91 ± 0.19^a^
B3	37.66 ± 2.64^b^	44.06 ± 2.39^b^	7.16 ± 0.26^b^	5.36 ± 0.31^ab^
B5	41.33 ± 2.51^c^	48.70 ± 1.68^c^	8.43 ± 0.32^c^	5.08 ± 0.21^ab^
B7	42.20 ± 1.52^c^	45.16 ± 1.37^b^	7.33 ± 0.14^b^	4.34 ± 0.17^b^

Values are expressed as mean ± SEM (*n* = 15). Mean values in the same column with different superscript letters vary significantly (*P* < 0.05).

**Table 6:** Superoxide dismutase (SOD) and malondialdehyde (MDA) activities observed on different sampling days after feeding *Labeo rohita* with banana peel flour (BPF) supplemented diets.

Diet	Immune response			
	SOD activity (unit mL^−1^)		MDA (nmol mL^−1^)	
	30 days	60 days	30 days	60 days
Control	43.1 ± 1.04^a^	44.5 ± 0.78^a^	9.16 ± 0.29^a^	8.24 ± 0.26^a^
B1	43.83 ± 0.88^a^	47.3 ± 0.62^ad^	8.83 ± 0.43^ab^	7.61 ± 0.43^ab^
B3	47.36 ± 0.65^ab^	49.93 ± 0.91^bd^	7.71 ± 0.26^bc^	7.12 ± 0.32^ab^
B5	49.28 ± 0.95^b^	52.5 ± 0.78^b^	7.23 ± 0.31^c^	6.66 ± 0.24^b^
B7	49.2 ± 1.19^b^	48.7 ± 0.53^cd^	7.56 ± 0.19^cb^	7.32 ± 0.44^b^

Values are expressed as mean ± SEM (*n* = 15). Mean values in the same column with different superscript letters vary significantly (*P* < 0.05).

**Table 7:** Catalase (CAT) activity and glutathione peroxidase (GPx) level measured on different sampling days after feeding *Labeo rohita* with banana peel flour (BPF) supplemented diets.

Diet	Immune response			
	CAT activity (unit mL^−1^)		GPx (unit per mg^−1^ of protein)	
	30 days	60 days	30 days	60 days
Control	13.43 ± 0.64^a^	14.07 ± 0.48^a^	15.46 ± 0.87^a^	15.87 ± 0.23^a^
B1	14.17 ± 0.81^ab^	14.83 ± 0.62^ab^	16.83 ± 0.57^ab^	17.31 ± 0.54^a^
B3	14.63 ± 0.52^ac^	16.52 ± 0.46^bc^	19.6 ± 0.64^bc^	17.06 ± 0.81^a^
B5	16.41 ± 0.67^bc^	17.8 ± 0.83^c^	21.04 ± 0.72^c^	16.54 ± 0.47^a^
B7	15.82 ± 0.79^c^	15.2 ± 0.61^ca^	21.36 ± 0.92^c^	15.72 ± 0.91^a^

Values are expressed as mean ± SEM (*n* = 15). Mean values in the same column with different superscript letters vary significantly (*P* < 0.05).

## Additional Points

Venkatachalam Sukumaran present address is Department of Zoology, Kundavai Nachiyar Government Arts College for Women (Autonomous), Thanjavur, Tamil Nadu 613007, India.
